# RNA virus-encoded microRNAs: biogenesis, functions and perspectives on application

**DOI:** 10.1186/s41544-020-00056-z

**Published:** 2020-10-12

**Authors:** Shoubin Zhan, Yanbo Wang, Xi Chen

**Affiliations:** Nanjing Drum Tower Hospital Center of Molecular Diagnostic and Therapy, State Key Laboratory of Pharmaceutical Biotechnology and Department of Physiology, Jiangsu Engineering Research Center for MicroRNA Biology and Biotechnology, NJU Advanced Institute of Life Sciences (NAILS), School of Life Sciences, Nanjing University, 163 Xianlin Avenue, Nanjing, 210023 China

**Keywords:** RNA virus, miRNA, Biogenesis, Biological function

## Abstract

MicroRNAs (miRNAs) are small, noncoding RNAs that regulate gene expression at the posttranscriptional level and play a crucial role in development and many diseases. The discovery of miRNAs has greatly expanded our understanding of the intricate scenario of genome-wide regulation. Over the last two decades, hundreds of virus-encoded miRNAs have been identified, most of which are from DNA viruses. Although the number of reported RNA virus-derived miRNAs is increasing, current knowledge of their roles in physiological and pathological processes has remained lacking. In this review, we discuss the biogenesis and biological functions of RNA virus- encoded miRNAs and their proposed roles in virus-host interactions and further underscore their potential value in the diagnosis and treatment of viral diseases.

## Background

### Discovery, biogenesis and functions of miRNAs

MicroRNAs (miRNAs) are a class of small, single-stranded noncoding RNAs of approximately 22 nucleotides (nt) in length that can regulate the expression of a target gene at the posttranscriptional level [1–3]. Since the discovery of the first miRNA, lin-4, 27 years ago [4], numerous miRNAs have been identified in animals, plants and viruses [5, 6] and found to play important roles in development and disease.

Canonical miRNAs are mainly transcribed by RNA polymerase II (Pol II) to generate the primary miRNA transcripts (pri-miRNAs) from either the protein-coding region or the noncoding region of the genome [7]. Pri-miRNAs are usually more than one kilobase long with stem-loop structures inside [8]. Pri-miRNA can be cleaved by the nuclear RNaseIII Drosha to generate ~ 60–70 nt precursor miRNA (pre-miRNA) hairpin followed by export to the cytoplasm where RNaseIII Dicer further processes pre-miRNAs into ~ 22 nt double-stranded RNAs [9]. The guide strand (mature miRNA) of the miRNA duplexes is then loaded into the RNA-induced silencing complex (RISC), whereas the other strand is degraded [10, 11].

The miRNA-loaded RISCs (miRISCs) bind the 3′ untranslated region (3′ UTR) of mRNAs and direct target transcripts repression [12]. As miRNAs and their targets are not paired one to one, a single miRNA can target multiple sites of transcripts and a single gene can be targeted by several miRNAs; miRNAs and their targets are involved in a complex regulatory network [6, 13].

### Virus-host interaction and microRNAs

Viruses, as organic species between living and nonliving organisms, engage in intracellular obligatory parasitism. They complete their whole life cycle within host cells by exploiting host biosynthetic machinery. As viruses can be thought of as transposons that constantly travel through hosts [14, 15], it is easy to consider the existence of coevolution between viruses and their hosts. Furthermore, following virus infection, host cells will activate their defense system to repress viral survival and replication, and viruses are also destined to respond by evolving certain mechanisms. Compared with proteins and long transcripts encoded by viral genes, miRNAs are more nonimmunogenic and flexible for viruses to specifically restrain host cell defenses and further establish a cellular environment that is conducive to viruses [16].

In the year 2004, Pfeffer and colleagues found the first miRNA encoded by Epstein-Barr virus (EBV), shedding light on the close relationship between miRNAs and viruses [17]. To date, 530 viral mature miRNAs have been identified from several viral families, such as herpesvirus, polyomavirus, papillomavirus and retroviruses [18, 19] according to miRBase [20]. The majority of viral miRNAs found are encoded by DNA viruses, predominantly in the herpesvirus family, and they have already been reviewed in detail for their biogenesis, functions and biological roles in host interactions as well as diseases [12, 15, 16, 21]. Furthermore, miRNAs encoded by viruses with RNA genomes have been recorded in an increasing number of studies; nevertheless, they have not been systematically reviewed. Here, we list the miRNAs encoded by diverse RNA viruses that include but are not limited to retroviruses (Table 1) and then summarize their biological functions. We also discuss the potential application values of these RNA virus-encoded miRNAs in the clinic.

**Table 1:** RNA virus-encoded miRNAs and their proposed functions

Virus family	Virus species	miRNA (s)	Target	Predicted function	Ref
Retroviridae	HIV-1	miR-N367	Nef	Viral replication inhibition	[18]
		vmiR88, vmiR89	TLR8^a^	Chronic immune activation	[22]
		miR-H3-3p	TATA box in 5′ LTR	Viral replication promotion	[23]
		TAR-derived miRNAs	TAR	Viral replication inhibition	[24, 25]
			IER3, ERCC1	Host cell apoptosis inhibition	[26]
			Aiolos, NPM/B23 Caspase 8, Ikaros	Host cell apoptosis inhibition	[27]
	BLV	miR-B4-3p	HBP1, PXDN^b^	Host cell neoplasia induction	[19, 28]
	ALV-J	E (XSR) miRNA			[29]
	BFV	miR-BF1-5p, miR-BF1–3p, miR-BF2-5p			[30]
Orthomyxoviridae	IAV H5N1	miR-HA-3p	PCBP2	Cytokine production enhancement	[31]
Flaviviridae	WNV	KUN-miR-1	GATA4	Viral replication promotion	[32]
	DENV-2	DENV–vsRNA-5	NS1	Viral replication inhibition	[33]
Filoviridae	EBOV	miR-VP-3p			[34]
		Zebov-miR-1-5p	Importin-α5	Host immune system evasion	[35]

^a^TLR8:vmiR88 and vmiR99 function by directly binding to TLR8 rather than loading into RISCs

^b^PXDN was confirmed as the target of miR-B4-3p by Kincaid, but significant down-regulation was not observed in ovine tumors

## Main text

### Nuclear RNA virus-encoded miRNAs

A virus with an RNA genome is named an RNA virus, which depending on the type of genome can be subdivided into double-stranded RNA (dsRNA), positive-sense single-stranded RNA (ssRNA^+^) and negative-sense single-stranded RNA (ssRNA^−^). Various families of RNA viruses exist, including Retroviridae, Orthomyxoviridae, Filoviridae, Flaviviridae, and Coronaviridae. Until now, miRNAs have been identified mainly from HIV-1, BLV (retrovirus), H5N1 (orthomyxovirus), EBOV (filovirus), WNV and DENV-2 (flavivirus), as discussed further below.

Nuclear RNA viruses, including retroviruses and orthomyxoviruses, complete transcription and replication of their genomes in the nucleus of host cells, which is different from other RNA viruses. Retroviruses are a group of ssRNA^+^ viruses that are reverse-transcribed into DNA and then integrated into the host genome to establish a persistent infection. To date, the large majority of RNA viruses identified to encode miRNAs are retroviruses [36].

#### MiRNAs encoded by human immunodeficiency virus type 1 (HIV-1)

After the identification of miRNAs from the herpesvirus family, many efforts have been made to investigate whether other virus families also encode miRNAs [37]. The possibility of the existence of HIV-encoded miRNAs has been examined by different groups via computer-directed analyses. Bennasser et al. proposed 10 extrapolated mature miRNAs within 5 candidate pri-miRNAs [38], and another screen was performed to find potential HIV-1 miRNAs and their targets in host cells [39]. Both of the bioinformatic predictions above reveal multiple host transcripts that could be regulated by the candidate HIV-1 miRNAs.

The first HIV-1 miRNA, named miR-N367, was experimentally identified in HIV-1-infected MT- 4 T cells by Omoto et al. in 2004 [18]. MiR-N367 is derived from the Nef gene [40] and is mainly associated with the early-stage replication of HIV-1 in host cells. MiR-N367 was recently confirmed by the same group to target the negative-response element (NRE) of the 5′ long terminal repeats (LTR) in vitro, which revealed a self-regulation mode for HIV-1 to repress its replication and maintain long-term nonprogressor (LTNP) states [41]. Shortly after that, a miRNA-like vshRNA1 was found in the Env region of HIV-1 with the ability to target Env without a detectable short precursor. In another instance, Zhang and colleagues employed deep sequencing combined with silico prediction and Northern blotting and identified miR-H3-3p and its possible precursor derived from the reverse transcriptase region of HIV-1 [23]. MiR-H3-3p was found to have high conservation among HIV-1 subtypes and a special ability to elevate the association between the TATA box and transcription factors by targeting the aforementioned motif of 5′ LTR, which could increase HIV-1 replication and reduce viral latency.

The trans-activation responsive (TAR) element, which forms a hairpin structure of ~ 50 nucleotides in length, is expressed during virus infection and can highly activate HIV-1 gene expression. An miRNA fragment with homology to the 5′ stem of the TAR element was detected by Klase et al. via RNase protection assays (RPA). The TAR stem-loop can be recognized by recombinant Dicer and processed into an miRNA in vitro. The author also speculated the ability of the TAR-derived miRNA to repress HIV-1 replication and further play a potential role in latency maintenance [24]. They later extended the functions of the TAR miRNAs by analyzing the HIV-1-infected gene expression of host cells via microarray and found that TAR miRNAs can repress multiple cellular genes related to apoptosis such as IER3 and ERCC1, which may be a mechanism for HIV-1 to prevent cell death and promote its survival [26]. Similar results have also been reported by another independent group. Ouellet et al. identified two other TAR-derived miRNAs named miR-TAR-3p and miR-TAR-5p in HIV-1-infected primary human CD4^+^ T lymphocytes [42], and the derived miRNAs could regulate apoptosis through modulating the caspase 8, Aiolos, Ikaros and NPM/B23 proteins of infected host cells [27]. Subsequently, Harwig et al. employed SOLiD deep-sequencing and discovered that miRNAs derived from the 3′ side of the TAR are loaded into RISC [43], which is consistent with the results from Ouellet and Li [25, 27]. Additionally, TAR-derived miRNAs and vmiR88/89 were identified by Narayanan [44] and Bernard et al., respectively [22], in exosomes derived from HIV-1-infected cells as well as the serum of HIV-positive people. These results, along with other reports, where viral miRNAs were found in exosomes during Epstein-Barr virus infection [45, 46], suggest that viral miRNAs in exosomes from infected cells could play a role in the intercellular spread of viruses and a potential biomarker for virus detection.

Other researchers employing high-throughput sequencing also proved the existence of the aforementioned HIV-1-derived miRNAs including TAR-derived and Nef-derived miRNAs with different abundance [47]. Yeung et al. detected 3 × 10^3^ copies of TAR-derived miRNAs and 61 copies of Nef-derived miRNAs per cell infected with HIV-1 [48], demonstrating along with the aforementioned evidence that TAR miRNAs account for the majority of HIV-1-encoded miRNAs while miRNAs derived from other regions also exist and are involved in several viral as well as host cell functions.

#### MiRNAs encoded by bovine leukemia virus (BLV)

Although a number of HIV-1-derived miRNAs are presented, controversy somehow exists because of the following reasons: first, the stem-loop structure of the potential miRNA precursor lying in the genome and transcripts of an RNA virus will probably increase the risk of being impaired by Drosha and Dicer; on the other hand, the miRNAs from HIV-1 show a relatively low copy number except those from TAR, which may result in less significant functions physiologically [48, 49].

An elaborate study was conducted by Kincaid et al. who considered the noncanonical miRNA biogenesis pathway and established an algorithm based on sequences of miRNA precursor and RNA polymerase III (Pol III) promoter and terminator to predict retrovirus-encoded miRNAs [19]. MiRNAs from five precursors were identified by Northern blotting and high-throughput sequencing in several BLV-infected cell lines. They further found that these miRNAs are well conserved, and one of them, miR-B4-3p, has an overlapping seed sequence with cellular miR-29. They suggested a Pol III-transcribed manner of miR-B4-3p generation because of the observation that pre-miRNA was not affected when Pol II was blocked. Researchers then conducted dependent tests and found that the level of miR-B4-3p pre-miRNA was not influenced when Drosha was repressed by siRNAs or when its activity was enhanced in vitro, confirming that miR-B4-3p was processed in a Dicer-dependent and Drosha-independent manner combined with the observation that pre-miRNA was increased by Dicer expression. Moreover, the stem-loop structure lying in the miRNA-coding region of BLV was identified to have the ability to protect the transcripts from Drosha cleavage, which may enable the RNA viruses that encode candidate miRNAs to maintain the stability of their genomes and transcripts to a certain extent. A consistent observation published by Rosewick and colleagues in the same month extended the understanding of BLV-encoded miRNAs by revealing that their generation is independent of the mRNA expression of BLV, and these miRNAs could also be found in BLV-infected sheep [28]. Both Kincaid and Rosewick predicted peroxidasin homolog (PXDN) and HMG-box transcription factor 1 (HBP1) as the targets of miR-B4-3p, yet only HBP1 was significantly downregulated in malignant B cells, pointing to the possibility that miR-B4-3p functions by repressing HBP1.

#### MiRNAs encoded by other retroviruses

MiRNAs have been identified in other retroviruses, such as avian retrovirus and bovine foamy virus, by deep sequencing. Yao and colleagues found a conserved miRNA derived from the E (XSR) element of avian leukosis virus subgroup J (ALV-J) and confirmed its generation by the canonical miRNA biogenesis pathway [29]. MiRNAs derived from bovine foamy virus (BFV) were discovered by Whisnant et al. also via deep sequencing analysis [30]. According to a sequencing analysis of chronically BFV-infected cells, miR-BF1-5p, miR-BF1–3p and miR-BF2-5p accounted for over 70% of the total small RNA reads, which is much higher than the most abundant cellular miR-21. In addition, three BFV miRNAs were derived from one Pol III-transcribed pri-miRNA containing two ~ 55 nt stem-loop structures and located in the 3′ LTR of BFV. They also showed evidence that miR- BF1-5p and miR-BF2-5p were present in peripheral blood leukocytes (PBLs) from calves infected by BFV for 6 months, demonstrating the in vivo presence of BFV miRNAs.

#### MiRNAs encoded by influenza A virus (IAV)

Influenza A virus is a negative-stranded RNA virus and a member of orthomyxoviridae with a ssRNA-genome that replicates and transcribes its genome in the nucleus without a DNA stage, similar to retroviruses. Varble et al. suggested the theoretical possibility that influenza A virus can encode miRNA via incorporation of cellular miR-124 hairpin, resulting in considerable expression of mature miRNA [50]. Of late, our group examined influenza A virus H5N1-infected cells and found a virus-encoded miRNA, miR-HA-3p, which was named for its location in the 3p arm of the HA segment [31]. We demonstrated by using Ago2-knockout cells that Ago2 but not Drosha or Dicer plays a dominant role in miR-HA-3p production. Given that H5N1 infection can disrupt the release of chemokines and proinflammatory cytokines, leading to a ‘cytokine storm’ and lung damage [51–54], correlations between viral miRNA and innate immune responses, especially cytokine production during H5N1 infection, were tested. Poly(rC)-binding protein 2 (PCBP2) was then validated to be regulated by miR-HA-3p posttranscriptionally. It is known that PCBP2 can downregulate the RIG-I/MAVS-mediated signaling pathway and lead to suppression of the cellular inflammatory response to viral infection [55]. We further discovered that PCBP2 was significantly elevated, and cytokine production, such as that of TNF-α, IFN-β, IL-1β and IL-6, was retarded when miR-HA-3p was blocked in both monocyte-derived macrophages (MDMs) and H5N1-infected mice. By blocking miR-HA-3p, we observed that lung inflammation in a mouse model was remitted, and the survival rate was also improved, revealing a potential therapeutic strategy based on silencing viral-encoded miRNA.

### MiRNA derived from cytoplasmic RNA virus

Unlike nuclear RNA viruses, cytoplasmic RNA viruses spend their whole life cycle in the cytoplasm of host cells without a DNA intermediate, and they are considered the least likely to encode bona fide miRNAs because of both a lack of access to classical miRNA microprocessors in the nucleus and the threat of genome destabilization. Nonetheless, cytoplasmic RNA viruses such as tick-borne encephalitis virus (TBEV) and Sindbis virus, which were artificially modified through insertion of an exogenous miRNA hairpin, were reported to express functionally mature miRNAs without interfering with viral replication or expression of essential viral proteins [56, 57]. Thus, natural cytoplasmic RNA viruses would also employ certain methods to circumvent these potential barriers, and several viruses including Ebola virus (EBOV), West Nile virus (WNV) and Dengue virus 2 (DENV-2) have been verified to encode miRNAs.

#### MiRNAs encoded by WNV and DENV-2

MiRNAs from WNV and DENV were reported by the same group. These two cytoplasmic RNA viruses with a ssRNA^+^ genome belong to the same flavivirus family, including a large class of arboviruses threatening the health of humans and animals worldwide. WNVKUN, a strain of WNV, was studied, and the production of KUN-miR-1 from the 3’UTR of WNVKUN was verified by Hussain and colleagues [32] in the first report, to our knowledge, of miRNA derived from natural cytoplasmic RNA viruses. KUN-miR-1 was proven to be processed by Dicer-1 and only identified in several mosquito C6/36 and Aag2 cell lines infected with WNVKUN but not mammalian cells, which can be explained by the tissue-specific expression of miRNAs. In vitro experiments further confirmed that KUN-miR-1 could facilitate viral replication through targeting GATA4; however, this function was achieved by elevating GATA4 mRNA, which differs from the normal miRNA function pathway. They identified DENV-vsRNA-5 from both mosquito cells and mammalian Vero cells infected with DENV-2, a serotype of DENV. AGO2 was suggested to engage in the biogenesis of DENV-vsRNA-5 via an immunoprecipitation (IP) analysis. DENV-vsRNA-5 can directly degrade the transcripts of viral nonstructural protein 1 gene (NS1) and trigger viral replication inhibition.

#### EBOV-encoded miRNAs

Ebola virus disease (EVD) is a severe epidemic disease caused by EBOV infection. During the 2014–2016 Ebola outbreak in West Africa, a total of 28,616 cases were reported with more than 11,000 deaths according to the World Health Organization [58]. Evidence that EBOV encodes miRNAs was proposed by Liang et al. for the first time [59]. Concentrating on the underlying diagnosis value of EBOV miRNA, the authors screened three putative miRNAs and selected miR- VP-3p located in the VP40 region because of its high conservation among EBOV strains [34]. Northern blotting detected miR-VP-3p in the exosomal fractions of sera from EBOV-infected patients. Data from a TaqMan probe-based qRT-PCR assay showed that the expression level of miR-VP-3p could not only distinguish patients in acute phase EBOV from those in recovery phase but also identify EBOV-positive patients earlier than their EBOV genomic RNA becoming positive. These findings provide new insights into the role of viral miRNA in early EBOV detection as well as the likely bioactivity during the virus infection period. Since then, more EBOV-derived miRNAs such as Zebov-miR-1-5p and miR-T1-5p have been predicted and explored by different groups [35, 60, 61]. Although discrepant observations of the expression levels of miRNAs were suggested to be caused by diverse testing techniques [61], together with all the studies mentioned above, we can still conclude that bona fide miRNAs are sufficiently encoded by EBOV and released by viral infected host cells, making cell-free EBOV miRNA a potentially powerful tool in the early diagnosis of virus infection.

## Conclusions

Until now, miRNAs discovered from RNA viruses, including retrovirus, orthomyxovirus, flavivirus and filovirus, have fewer species and lower abundance than those from DNA viruses. Among over 500 viral miRNAs recorded by miRBase [20], only approximately 30 are mature RNA virus- encoded miRNAs. The discovery of these viral miRNAs add more insights into our knowledge of relatioship between viruses and miRNAs, however, pitfalls still exist in current studies. Results from some studies failed be replicated by following deep sequencing or by different experimental methods. Due to low copy number of some RNA-derived miRNAs, different extraction and verification protocols could have a considerable impact on the qualitative and quantitative data of miRNAs. Also, controversies still exist partly due to several barriers such as cytoplasmic RNA virus having no access to the whole miRNA processing machinery and viral genome instability induced by endonuclease that can recognize the miRNA-encoding stem-loop structure [62]. Nevertheless, some evidence shows that some RNA viruses will take advantage of the noncanonical biogenesis pathway in host cells to generate their own miRNAs, and certain stem-loop structures within their genomes and transcripts are likely resistant to RNase digestion [19]. The fact that several groups have failed to find the aforementioned miRNAs actually raise doubts about their authenticity [63, 64]; thus, a well-established methodology combining multiple computational and experimental analyses must be utilized to yield reliable and reproducible results.

Whether there is a general mechanism of biogenesis and biological functions of RNA virus-derived miRNAs remains unclear. According to existing reports, RNA viruses will employ various RNA endonucleases, including Dicer and Ago, to catalyze maturation of their miRNAs, and not all viral miRNAs identified have been found with a valid target or certain functions. However, miRNAs encoded by viruses such as HIV-1 and H5N1, which have verified target and biological functions, will provide a deeper understanding of virus pathogenesis mechanism as well as novel therapeutic targets for drug development to treat these virus-caused diseases. Furthermore, considering the high mutation rate of RNA viruses, some of these miRNAs, especially those without high conservation among strains, may be a product of RNA virus mutation and evolution; however, more evidence supporting this concept remains to be explored.

A few RNA virus-derived miRNAs are also present in exosomes from host cells and stably exist in the serum and plasma of people infected with EBOV and HIV-1 [34, 44]. It is widely known that in multicellular organisms, miRNAs secreted by cells can reach remote tissues and organs to mediate their target genes as well as signaling pathways, and they have been shown to play a crucial role in multiple physiological and pathological processes [65–68]. Evidence suggests that exogenous miRNA originating from RNA viruses could also participate in intercellular communication, making bystander cells more susceptible to viral infection. Indeed, whether viral and endogenous miRNAs share the same packaging, secretion and function mechanism is still unknown. Moreover, RNA virus-encoded miRNA that is detectable before the viral genome becomes positive in serum and plasma may serve as a biomarker for EBOV and other viruses, which will help with promoting virus detection accuracy and advancing the diagnosable window. In this regard, it is worth asking if the range of RNA viruses that encode secreted miRNAs can be extended when more viruses are tested. Moreover, no miRNA has been identified from the newly discovered coronavirus SARS- CoV-2, which is causing the worldwide epidemic COVID-19, and the Middle East respiratory syndrome coronavirus (MERS-CoV), which causes MERS. However, studies on determining the existence and latent diagnosis value of these recently appeared coronaviruses are still ongoing.

Cumulatively, despite many examples during the last 20 years, current knowledge on RNA virus-encoded miRNAs is still lacking in contrast with their analogues from DNA viruses. Nonetheless, efforts that have been made to uncover the detailed mechanism of biogenesis, roles in virus-host interaction and possibility of being a biomarker will definitely provide priceless insights into this area.

## Data Availability

Not applicable.

## Abbreviations

miRNA: microRNA
Pol II: Polymerase II
Pol III: Polymerase III
pri-miRNA: Primary miRNA transcript
pre-miRNA: Precursor miRNA
RISC: RNA-induced silencing complex
3′ UTR: 3′ untranslated region
EBV: Epstein-Barr virus
dsRNA: Double-stranded RNA
ssRNA^+^: Positive- sense single-stranded RNA
ssRNA^−^: Negative-sense single-stranded RNA
HIV-1: Human immunodeficiency virus type 1
NRE: Negative-response element
LTR: Long terminal repeats
LTNP: Long-term nonprogressor
TAR: Trans-activation responsive
RPA: RNase protection assays
BLV: Bovine leukemia virus
PXDN: Peroxidasin homolog
HBP1: HMG-box transcription factor 1
ALV-J: Avian leukosis virus subgroup J
BFV: Bovine foamy virus
PBLs: Peripheral blood leukocytes
IAV: Influenza A virus
MDMs: Monocyte-derived macrophages
TBEV: Tick-borne encephalitis virus
EBOV: Ebola virus
WNV: West Nile virus
DENV-2: Dengue virus 2
NS1: Nonstructural protein 1 gene
EVD: Ebola virus disease
